# The Acoustic Properties of Low Intensity Vocalizations Match Hearing Sensitivity in the Webbed-Toed Gecko, *Gekko subpalmatus*

**DOI:** 10.1371/journal.pone.0146677

**Published:** 2016-01-11

**Authors:** Jingfeng Chen, Teppei Jono, Jianguo Cui, Xizi Yue, Yezhong Tang

**Affiliations:** Department of Herpetology, Chengdu Institute of Biology, Chinese Academy of Sciences, Chengdu, Sichuan, China; Virginia Commonwealth University, UNITED STATES

## Abstract

The design of acoustic signals and hearing sensitivity in socially communicating species would normally be expected to closely match in order to minimize signal degradation and attenuation during signal propagation. Nevertheless, other factors such as sensory biases as well as morphological and physiological constraints may affect strict correspondence between signal features and hearing sensitivity. Thus study of the relationships between sender and receiver characteristics in species utilizing acoustic communication can provide information about how acoustic communication systems evolve. The genus *Gekko* includes species emitting high-amplitude vocalizations for long-range communication (loud callers) as well as species producing only low-amplitude vocalizations when in close contact with conspecifics (quiet callers) which have rarely been investigated. In order to investigate relationships between auditory physiology and the frequency characteristics of acoustic signals in a quiet caller, *Gekko subpalmatus* we measured the subjects’ vocal signal characteristics as well as auditory brainstem responses (ABRs) to assess auditory sensitivity. The results show that *G*. *subpalmatus* males emit low amplitude calls when encountering females, ranging in dominant frequency from 2.47 to 4.17 kHz with an average at 3.35 kHz. The auditory range with highest sensitivity closely matches the dominant frequency of the vocalizations. This correspondence is consistent with the notion that quiet and loud calling species are under similar selection pressures for matching auditory sensitivity with spectral characteristics of vocalizations.

## Introduction

Vocal communication involves the transmission of information encoded in the acoustic features of calls by senders to receivers, and the subsequent use of that information by the receivers in social interactions [1]. Acoustic information transfer may be interfered with by noises occurring ubiquitously in natural environments. In the case of sexual vocal communication, correspondence between the spectral energy distribution of male acoustic signals and auditory tuning (called the “matched-filter hypothesis”) is generally expected since this can minimize signal attenuation and interference [2]. In vertebrates the matching hypothesis has received extensive support since its original formulation [3–8], especially in those species inhabiting environments characterized by high levels of biodiversity and acoustic complexity [9, 10]. Nevertheless, several instances of mismatches between acoustic signals and auditory sensitivity and/or behavioral preferences have also been reported [11–14]; the lack of strict correspondence in these cases has been explained in term of interactions between natural and sexual selection, environmental and sensory system constraints [15–18]. Therefore, the degree of concordance between acoustic sender and receiver characteristics may be determined by factors specific to each species.

Among squamate, geckos are known for possessing vocal cords which facilitate the production of complex vocalizations. Some geckos are known to call loudly in tropical and subtropical regions; thus a number of studies on gecko acoustic communication has focused on such high-amplitude, long-range vocalizations [19, 20]. Many studies have suggested that geckos use this type of call as advertisement signals directed toward unspecified conspecifics at long distances [17–23]. For instance, the Tokay gecko, *Gekko gecko* emits loud calls which can be perceived at more than 100 m by human observers [19]. Recently the focus of gecko research has expanded to low-amplitude vocalizations (quiet calls) emitted during social interactions within close proximity [24, 25]. For example, the Japanese house gecko, *G*. *japonicus* uses only quiet calls for intraspecific communication [25]. Despite interest in gecko vocalizations, species differences in the auditory sensitivity of this group have received relatively little attention, especially in quiet callers. Moreover the relationships between call frequency characteristics and the sensitivity of the peripheral auditory system are largely unknown in these species.

Single unit nerve recordings, cochlear microphonics and auditory brainstem responses (ABRs) can be used to examine peripheral auditory system sensitivity. ABRs are short latency microvolt potentials evoked by acoustic stimuli, consisting of waveforms having three to five peaks occurring within 10 millisecond of stimulus onset; the earliest ABR peak is generated by the auditory nerve, whereas peaks of longer latency originate in the brain stem and midbrain [26]. During the last two decades, ABR measurement has been increasingly used by researchers studying auditory physiology [6, 7, 27]. A major advantage of the ABR method is that it is only very slightly invasive because it involves the use of subcutaneous electrodes. Another advantage of the ABR method is that it is widely applicable having been used in diverse vertebrates including geckos [28–31], thus making it possible to compare data from species in different taxa.

The webbed-toed gecko, *Gekko subpalmatus* [32] is a common nocturnal gekkonid, mainly distributed in southern China [33, 34]. Because *G*. *subpalmatus* does not signal with advertisement calls over long distances, this has been regarded as a mute species [33]. However, because a closely-related species, *G*. *japonicus*, is known to communicate with quiet calls [25], we investigated the possibility that *G*. *subpalmatus* also produces quiet calls during social interactions. Thus, in the present study we first verified that *G*. *subpalmatus* produces quiet calls by recording vocalizations during male-female dyadic interactions. Secondly we used ABR measurements to determine the auditory sensitivity in order to determine if a matching pattern exists between vocalization characteristics and hearing sensitivity in this species.

## Materials and Methods

### Ethics statement

This study was conducted in accordance with a protocol from the Animal Care and Use Committee of Chengdu Institute of Biology (permission number: 20130720). Sodium pentobarbital (50 mg/kg) was used to anesthetize geckos for ABR tests and all subjects recovered within 2–3 hours. No specific permissions were required for animal collection in this area. All animal collection procedures were carried out in accordance with the Law of the People’s Republic of China on the Protection of Wildlife.

### Study subjects

All geckos were collected in urban areas in Chengdu, Sichuan, Southwestern China (30°37’ N, 104°05’ E). The geckos were housed individually in 15 × 20 × 15 cm plastic cages with free access to water and cockroaches (*Blatta lateralis*) dusted with vitamin supplements. Water spray was applied to the cage wall every 3–4 days. The ambient temperature of the room housing the geckos was maintained at 24 ± 1^°^C and the light: dark cycle was set to 14L: 10D light conditions (light: 0600–2000 h). We used 18 individuals (nine males and nine females) for call recording and 12 individuals of *G*. *subpalmatus* (seven males and five females) for auditory sensitivity tests. We chose geckos with snout-vent lengths (SVL) larger than 55 mm, which were considered sexually mature based on size in congeners, *G*. *japonicus* and *G*. *hokouensis* [35, 36], as subjects. All geckos were maintained in captivity for at least two weeks before the experiments. All trials were conducted between October 2013 and April 2014.

### Behavioral experiments and call recording

The behavioral encounter experiment was conducted at night (2100–0200 h) using an experimental cage measuring 50 × 25 × 20 cm. The cage was made of mat black acrylic plates, except for the top and front, which were made of transparent acrylic plates. The experimental cage was separated into two equal-sized compartments by a removable partition. One male and one female were introduced into the cage, one in each compartment, where each remained for more than 3 hours before each trial. The experiment was conducted in the dark with infrared light-emitting diode (LED) lamps (HVL-LEIR1; peak wavelength; 627 nm; Sony, Tokyo, Japan) set above the cage. Each trial began when the partition was removed from the cage allowing the geckos to interact. Gecko behaviors were recorded for 60 min with a digital camcorder sensitive to infrared light (HDR-PJ760; Sony, Tokyo, Japan) which was placed in front of the cage in the absence of observers. A portable PCM recorder (LS-10; frequency response; 70–20,000 Hz, OLYMPUS, Tokyo, Japan) placed inside the cage recorded all gecko calls produced in the cage during each trial. The cage was rinsed with water between trials to remove any gecko odor. If a male did not emit calls in a trial, the male was used on another trial. Several females were used in more than one trial. When a given gecko was used more than once, the intertrial interval was at least two weeks. We measured the maximum SPL of gecko vocalizations by playing back the calls recorded at 50 cm from the subjects and their amplitude was calibrated against a continuous tone of known SPL (50–70 dB SPL) recorded with the same recorder at the same recording level.

### ABR measurements

We conducted auditory tests between 0800 h and 1800 h. We weighted the geckos and then anesthetized them with an injection of barbiturate (50 mg/kg i.p.) [37]. After 5–10 min, we placed the geckos in a soundproof chamber whose ambient temperature was controlled at 27 ± 1 ^o^C with an electrical heater. We then inserted three needle electrodes (Rochester Electro-Medical, Inc. FL, USA) under the skin 1) behind the stimulated ear, 2) at the top of the head, and 3) in the ipsilateral forelimb. Responses to brief tone bursts, emitted through a portable amplified field speaker (SME-AFS, Saul Mineroff Electronic Inc, USA), placed 50 cm in front of the subjects, were evoked at frequencies between 0.2–7.0 kHz with intensity levels of 30 to 70 dB SPL (5 dB SPL interval). The entire procedure including stimulus presentation, ABR acquisition, equipment control, and data management was similar to our previous work [38]. Briefly, stimulus generation and ABR recordings were carried out using a digital processor RM2 (Tucker-Davis Technologies, Gainesville, USA) via fiber optic cables linked via RA4 and a USB linked to a computer running the custom software package OpenABR. Before ABR recording, acoustic stimuli levels were calibrated with a G.R.A.S 46BE 1/4 inch microphone (G.R.A.S Sound & Vibration, Denmark) with CCP Supply (Type 12AL, G.R.A.S) positioned at the location of the geckos head. ABR stimuli were delivered in random order with the frequency varying from 0.2 kHz to 7.0 kHz. The stimuli had 1-ms cos2 onset/offset ramps, 3 ms plateau time and sample rate of 24, 414 Hz. The presentation rate was 4/s. The stimulus presentation rate was similar to the pulse rate of natural vocalizations of *G*. *subpalmatus* and 5-ms duration was used because ABRs are not evoked with stimuli having longer durations, resembling the natural calls (unpublished data, Chen et al.). At each frequency, we recorded ABRs at nine intensity levels ranging from 70 down to 30 dB SPL in 5 dB SPL steps. Each ABR was the average response to 400 stimulus repetitions. All biological signals were notch filtered at 50 Hz during data collection.

### Data analysis and statistics

The paired geckos were easily distinguished from each other by watching the videos that recorded the entire trial period. The calls of gekkonids can be classified into single chirp and multiple chirp calls; multiple chirp calls are considered to be more essential to social interactions [25, 39]. For this reason we used multiple chirp calls for analysis. Females rarely call and therefore only male calls were used in acoustic analyses. Recorded calls were analyzed using Adobe Audition 3.0 (Adobe Systems, San Jose, CA, USA). Each call was digitized using the following parameters: a sampling rate of 48 kHz (16 bits), frame length of 512 samples, and Hamming window type. The structure of individual chirps in each call was analyzed, and the following variables were measured for each call: the numbers of chirps per call, call duration, mean chirp duration, mean inter-chirp interval, chirp rate, mean dominant frequency, maximum sound pressure level.

The ABR thresholds and latencies were determined using methods similar to those described previously [29]. Thresholds were defined as the lowest stimulus level at which a repeatable response could be recognized. Latencies were measured as the first ABR valley using manually placed cursors in Matlab (Version 2009a). Because there are no sex differences in the ABR thresholds and latencies of *G*. *subpalmatus*, the data of females and males were combined for the analysis. All data were sorted and analyzed using the SPSS 16.0 statistical program (SPSS Inc., Chicago, IL, USA). For all tests, data were expressed as mean ± SE.

## Results

### Call characteristics

**Fig 1:**
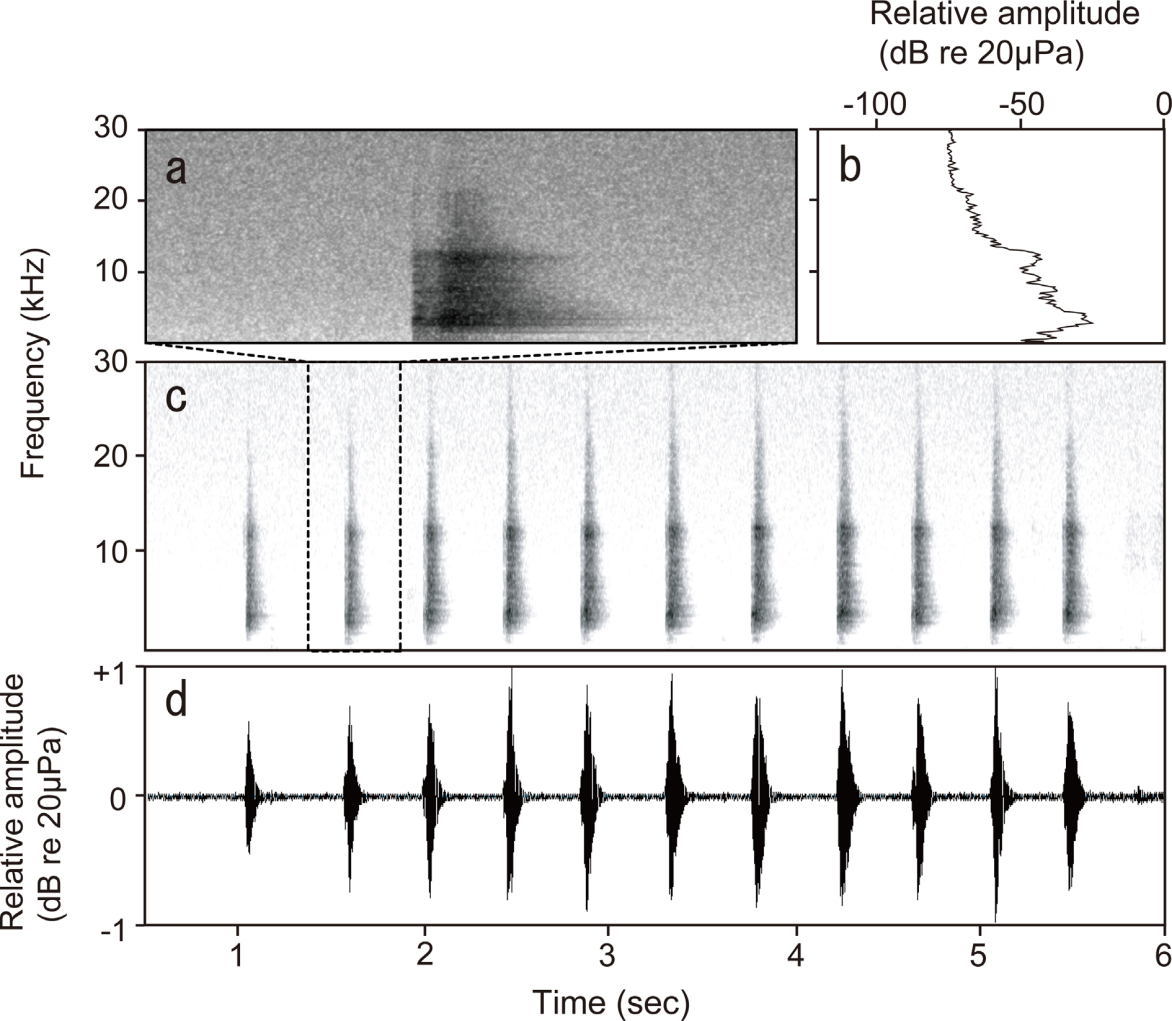
A typical call structure of *Gekko subpalmatus*. (a) An amplified sonogram and (b) power spectrum of one chirp from a male *Gekko subpalmatus* call. (c) Sonogram and (d) amplitude-modulated waveform of a male *Gekko subpalmatus* call.

We recorded quiet calls from *G*. *subpalmatus* during social encounters. In the encounter experiments, seven individuals emitted a total of 48 calls. All calls were too weak to be recorded by the digital camcorder at a distance of 2 m from the cage, thus the analyses of the call structures were based on the recordings obtained by a portable PCM recorder. A spectrogram of a typical *G*. *subpalmatus* male call is shown in Fig 1. The chirps consisted of multiple-frequency components and broadband spectral energy without any clear harmonics. Spectral analysis showed a dominant frequency range at 2.47–4.17 kHz with the average peak at 3.35 ± 0.06 kHz. Each call of *G*. *subpalmatus* consisted of 3–29 chirps (average: 12.69 ± 0.93) with a regular temporal pattern. Each call lasted 0.86–11.63 sec (average: 5.24 ± 0.39). The chirp rate and duration were 2.78–5.24/sec (average: 3.72 ± 0.07) and 0.08–0.13 sec (average: 0.12 ± 0.002), respectively. The range of maximum amplitude of the calls was 51.4–61.1 dB SPL (average: 54.6±1.6).

### ABR wave morphology

**Fig 2:**
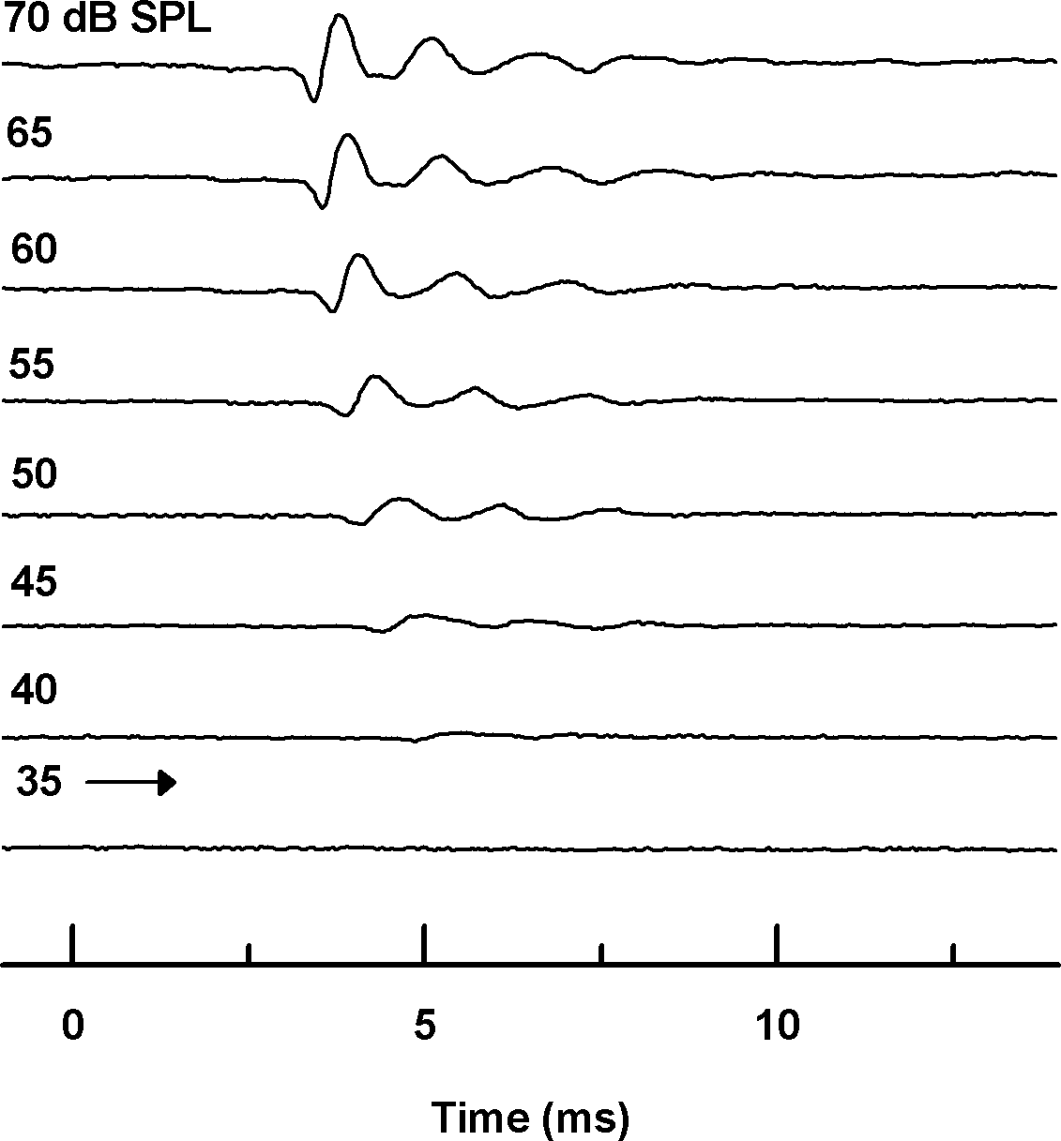
Representative ABR waveforms for a single gecko showing multiple waveform peaks within the first 5–10 ms in response to 3 kHz tone bursts ranging from 35 to 70 dB SPL.

**Fig 3:**
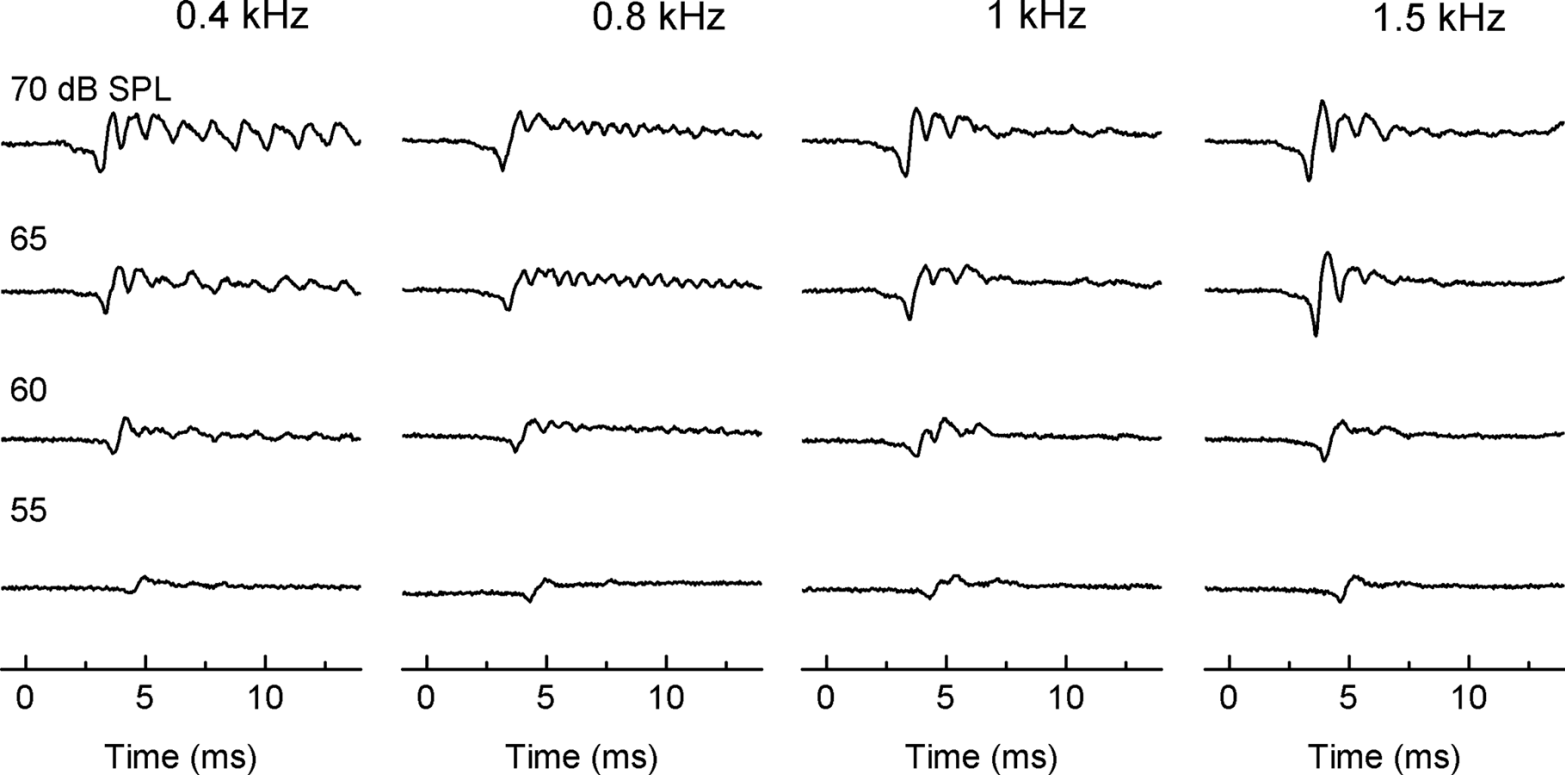
Response waveforms from 0.4 kHz to 1.5 kHz reveal a well-defined frequency following response (FFR) that masked late response peaks.

Since *G*. *subpalmatus* was found to produce quiet calls, we further measured ABRs. Similar to the ABR waveforms of other geckos, *G*. *subpalmatus* ABRs exhibited 3–4 potential peaks within 10 ms of stimulus onset (Fig 2). ABR amplitude increased as stimulus intensity increased above thresholds, whereas latency decreased. When *G*. *subpalmatus* was stimulated with low frequency (0.2 Hz–0.8 kHz) tone bursts at high intensity (~65 dB SPL, Fig 3), the frequency following response (FFR) was manifest. The FFR is a characteristic response pattern in which the evoked neural activity pattern is time locked to the temporal structure of the pure tone stimuli [30].

### ABR thresholds

**Fig 4:**
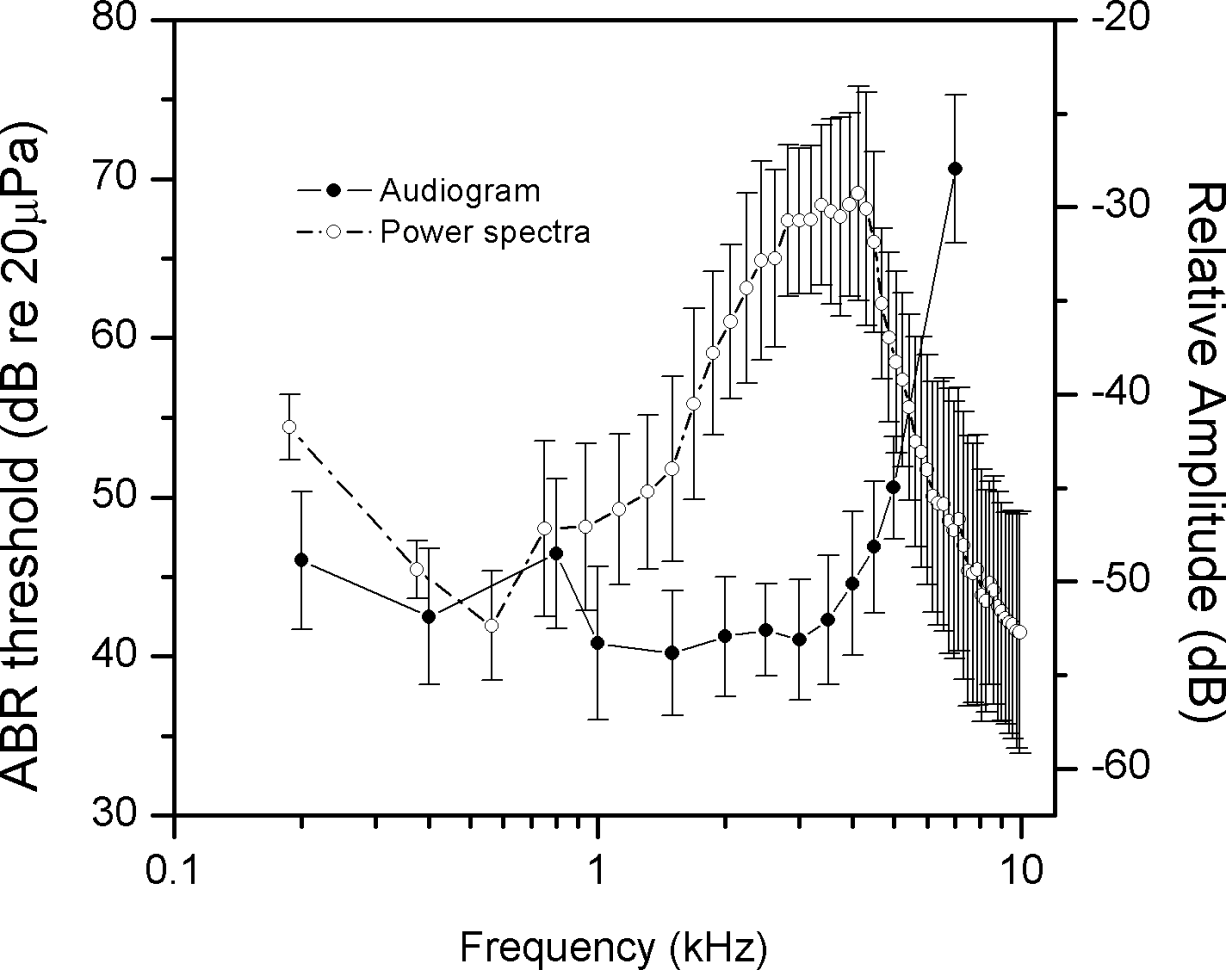
Audiogram and power spectra of vocalizations for *Gekko subpalmatus*(±SE).

The ABR threshold at a given frequency is the lowest stimulus intensity that evokes a detectable ABR. ABR thresholds were significantly affected by frequency (F_12, 132_ = 91.091, p < 0.001; Fig 4). ABR thresholds were lower from 1.0 to 3.5 kHz (~40 dB SPL) than at both higher (4 kHz, ~45 dB SPL) and lower frequencies (0.8 kHz, ~46 dB SPL). Thresholds increased significantly above 4.0 kHz, particularly from 5.0 to 7.0 kHz, where 20 dB SPL increments were observed. In addition, the geckos were relatively more sensitive within another narrow band (~0.4 kHz, ~42 dB SPL).

### ABR latency

**Fig 5:**
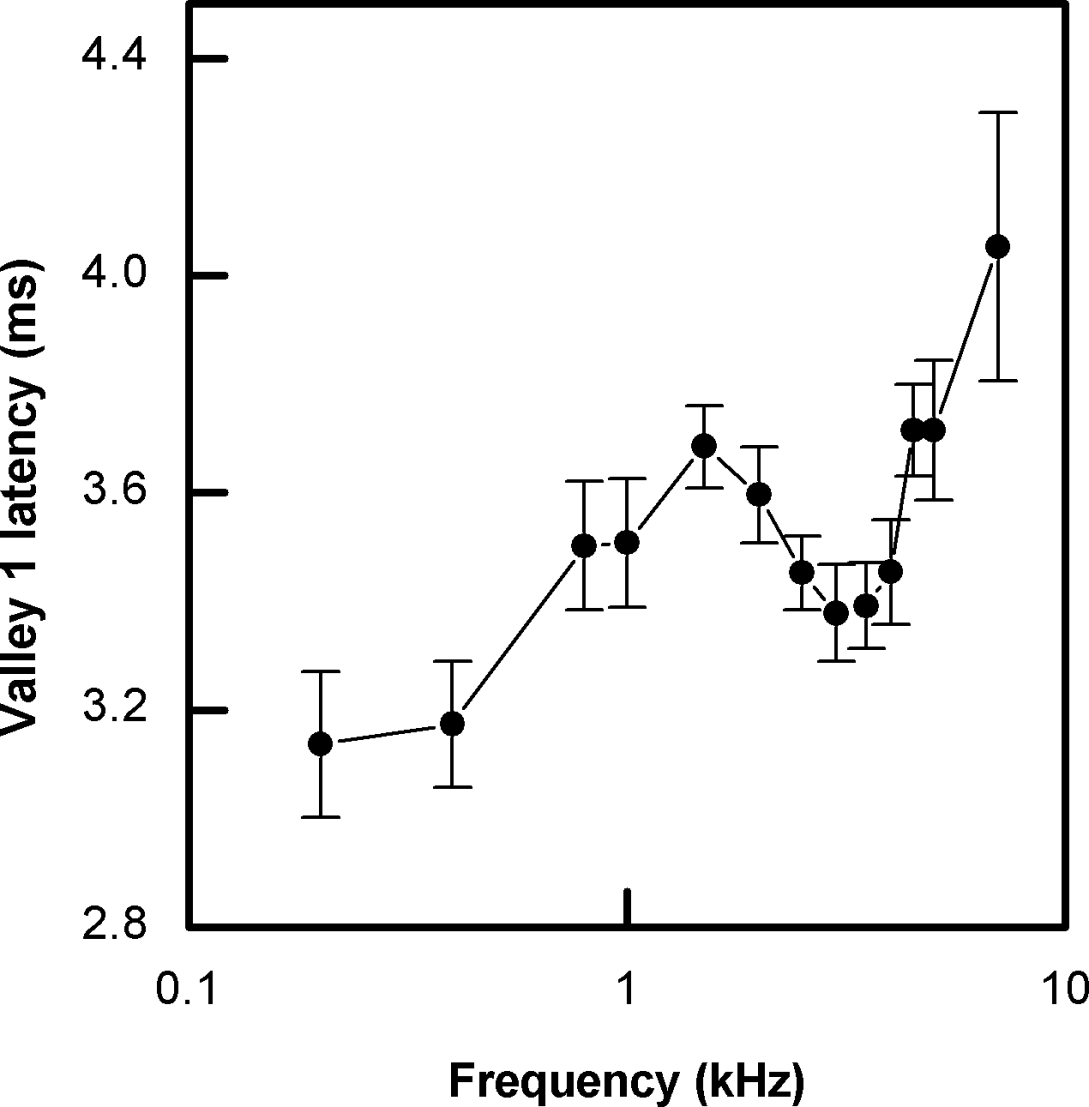
ABR latency of *Gekko subpalmatus* for tones of different frequencies at 70 dB SPL(±SE.

ABR latency is regarded as an additional indicator of auditory sensitivity. In general, ABR latency decreases as stimulus intensity increases above threshold [7]. The ABR latency pattern of *G*. *subpalmatus* was similar at different sound pressure levels. This is exemplified by variations in ABR latency at 70 dB SPL as a function of frequency (Fig 5). As shown in Fig 5, ABR latency was significantly affected by frequency at 70 dB SPL (F_11, 121_ = 80.807, p < 0.001). Note that two valleys of ABR latency are apparent for *G*. *subpalmatus* at 0.2–0.4 kHz (3.38–3.40 ms) and 3.0–4.0 kHz (3.41–3.38 ms); both of which partially overlap the frequency sensitivity range detected by ABR thresholds. Specifically ABR latency maxima occur at 1.5–2.0 kHz and for the frequency band beyond 4.5 kHz.

## Discussion

### Interspecific differences in temporal patterns of calls

In the present study, we found that male *G*. *subpalmatus* emit low amplitude calls, consistent with the idea that quiet calling involves many species in the genus *Gekko*. These calls likely function as contact calls insofar as low amplitude calls function as contact calls in a congener species *G*. *japonicus* [25]. *G*. *subpalmatus* calls are composed of multiple single chirps which are similar to the call structure of the gekkonids, *Hemidactylus frenatus*, *H*. *mabouia*, *Ptenopus garrulus*, and *G*. *japonicus* [21–23, 25]. Despite a close phylogenetic relationship there are differences in the temporal characteristics of calls between *G*. *subpalmatus* and *G*. *japonicus*. Thus the number of chirps per call is quite different between these species (12.69 in *G*. *subpalmatus*; 5.32 in *G*. *japonicus*), although some parameters such as peak frequency and chirp duration are similar. In view of the fact that there is partial overlap between the geographic distributions of these two species [33], it is possible that chirp repetition rates may serve for species recognition, as has been extensively reported for frogs and insects [5]. Further studies are needed to explore the functional role of the chirp repetition rate in *Gekko* calls.

### Correspondence between vocalization structure and hearing sensitivity

Geckos possess the largest basilar papillae among lizards, and the unique tonotopic organization of sensory cells on the basilar papillae determines the frequency range and resolution of the auditory system in these species [40]. Because behavioral assays of hearing sensitivity are difficult to carry out in relatively inactive reptilian species, several neurophysiological measurements such as ABR are now widely used to assess auditory thresholds and frequency sensitivity [41–43]. In general, gekkonids share a similar hearing frequency range of around 0.1–5.0 kHz with an upper frequency limit close to 7 kHz [44]. The results of ABR experiments show that the most sensitive frequency ranges of *G*. *subpalmatus* are 1.0–3.5 kHz and at 0.4 kHz. These frequency ranges are related to the two segments of the gecko papilla sensitive to frequencies approximately below and above 1 kHz [29]. Different studies show that ABR latencies are dependent on stimuli SPL and frequencies [7, 29]. The ABR latencies of *G*. *subpalmatus* increase at high frequencies, similar to the functional pattern reported in birds but in contrast with the extended high frequency sensitivity of the mammalian ear [7, 45]. This contrast is consistent with the closer relationship between lizards and birds in the current phylogeny of living tetrapods [46].

If the tuning curves obtained here based on both ABR latency and threshold are taken into account, a narrow range of best frequency sensitivity can be identified at 3.0–3.5 kHz. This peak sensitivity region corresponds to the mean dominant frequency (3.35 kHz) of the male calls of *G*. *subpalmatus*. In contrast the sensitivity peaks of the loud calling congener, *G*. *gecko*, are around 0.6 and 1.6 kHz [39]. The first peak corresponds closely to the dominant frequency of male *G*. *gecko* calls, around 0.5 kHz, while the second peak appears to match a secondary frequency peak in their calls [39, 47]. Thus in the cases of both weak (i.e. *G*. *subpalmatus)* and loud (*G*. *gecko*) callers, auditory tuning is closely matched with call design. It is notable that in fossorial pigopod gekko species, matched vocalization acoustics and auditory sensitivity extending into the ultrasonic range has been reported, although the amplitude of the vocalizations of these species was not specified [48]. Taken together these data support the idea that intraspecific communication can exert strong selection for matching auditory sensitivity to the acoustics of male vocalizations. Nevertheless this work is based on a limited number of species. Additional work including non-vocal gekkonids may provide important insights into the nature of the coupling between call signal characteristics and auditory preferences [5].

### Comparisons of auditory thresholds and the frequency following response (FFR) in lizards

Body size is positively correlated with auditory threshold sensitivity in gekkonids; this correlation may be partially explained by interspecific variations in ear structure [43]. The present results are consistent with this finding insofar as ABR thresholds in *G*. *subpalmatus* (40–43 dB SPL) are less sensitive than those of *G*. *gecko* (~30 dB SPL), one of the largest species in *Gekko*. In addition, the maximum SPL of *G*. *subpalmatus* calls estimated at 50 cm away from the sound source is 54.6 dB SPL, implying that geckos may communicate over longer distances, implying that these signals convey territory information.

In general, the phase-locking of frequency following responses (FFRs) will degrade with increasing frequency, reflecting the low-pass nature of brainstem phase locking [49]. For humans, FFRs can be tracked to frequencies as high as 1.5 kHz; for geckos, FFRs can be recorded up at 0.4 kHz and 0.8 kHz in *G*. *gecko* and *G*. *subpalmatus*, respectively. FFRs have only rarely been found in other lizards (e.g. green anole, *Anolis carolinensis*) which are non-vocal species [29, 46]. FFR variations in lizards may reflect anatomical or physiological differences in the auditory organ. However, *G*. *gecko* is the only gecko species in which the basilar papilla has been studied in detail. Thus identifying relationships between FFRs and variations in the structure of the *Gekko* ear is not possible at this time.

In summary, the results of the present study show that male *G*. *subpalmatus* produce quiet or low amplitude calls, and that receiver's auditory spectral sensitivity is closely matched with the range of the dominant frequency of their signal. These results support the idea that intraspecific communication has been an important selective force in the evolution of auditory sensitivity in this species. It is also possible that the temporal component of the chirps in the calls of this species might function for species recognition.
