# Correction: The Acoustic Properties of Low Intensity Vocalizations Match Hearing Sensitivity in the Webbed-Toed Gecko, *Gekko subpalmatus*

**DOI:** 10.1371/journal.pone.0170831

**Published:** 2017-01-24

**Authors:** Jingfeng Chen, Teppei Jono, Jianguo Cui, Xizi Yue, Yezhong Tang

There is an error in Fig 4. According to the suggestion of Dr. Geoffrey Manley (personal communication), when the rise time of the ABR stimulus is shorter than one cycle of the stimulus (for instance, the stimuli at 0.2 kHz have a rise time of less than 5 ms), the stimulus will contain spectral splatter and this spectral splatter will provide energy at higher frequencies, leading to an inaccurate threshold measurement. In the system described in this manuscript, the rise time was constantly set to 1 ms. Therefore, the points below 1 kHz have been omitted in the corrected Fig 4. Please see the corrected Fig 4 here.

**Fig 4:**
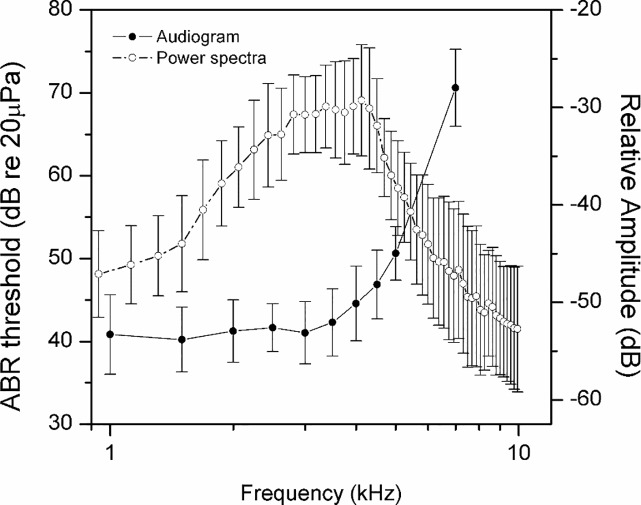
Audiogram and power spectra of vocalizations for *Gekko subpalmatu*s(±SE).

There is an error in the sixth sentence of the second paragraph under the subheading “Correspondence between vocalization structure and hearing sensitivity” in the Discussion section. The correct sentence is: It is notable that in fossorial pigopod gekko species, matched vocalization acoustics and auditory sensitivity extending into the ultrasonic range has been reported. The report showed that at higher frequencies, the increase in the sound pressure of the vocalizations matched the rise of thresholds across the same frequency range, such that effectively, the sound level perceived by the animals stayed relatively constant across a broad range of frequencies.
